# Special Richmond and Goslee Crowns

**Published:** 1915-07

**Authors:** Ardashes J. Nishon, Eber J. Reynolds


### SPECIAL RICHMOND AND GOSLEE CROWNS.

READ BEFORE THE STUDENTS' DENTAL SOCIETY OF THE UNIVERSITY OF MICHIGAN, MAY 17, 1915.

BY ARDASHES J. NISHON AND EBER J. REYNOLDS.

The subject of "Crowns" as you all know, is a broad one. Volumes of books have been written upon this subject, and we do not think that any one will expect us to cover the whole ground in the few minutes that we have. We have endeavored to cover that part of the subject which will be more interesting and may have more of practical value for us. In our knowledge of crowns there are no other crowns which require so much skill and accuracy of technique as the ordinary Richmond and Goslee crowns. By saying skill, we especially refer to the diagnosis of the case and the bringing out of such facts as will lead us to insert the most desirable crown. We also want to say a few words about devitalizing pulps, temporary crowns, and a few other points which apparently may seem commonly known, but which still should be of some value to us.

Before we concentrate our thought on the subject proper, it may be interesting for us to give something of the history and development of crowns. As you already know, the attempt made to reconstruct broken-down teeth with artificial crowns is as old a history as the art of dentistry. But constructions which may be called crowns from our standpoint, were made in the eighteenth century, and only fairly well developed at the last half of the nineteenth century. The tendency during all this period was to put this work on an artistic basis in the broadest sense of the word. So called ''Pivot Teeth'' described in Fuchard's work published in 1728, were carved in ivory or were natural crowns with silver or gold posts. The roots were first filled with metallic lead points, holes were then drilled for the post, which was cemented in. In 1816 the so called ''Mineral Teeth'' (porcelain) came out, but it was not until 1840 that any attention was given to the actual preservation of the roots. The crowns were misfitted and soon became loose. Without investigating the real cause of the trouble, they thought that the wooden post would serve the purpose better on account of expanding in saliva, and forming a tight joint, and also be easily removed for the treatment of possible abscesses. At this time various forms of posts with various forms of root preparations were used, as for example, Foster and Mack crowns. In 1873 the telescope crowns came into existence by Beer, or as claimed, by Morrison, a few years ago, which proved more satisfactory in the preservation of the root. Several other forms of porcelain crowns with their special forms of root preparations made their appearance during this period. Finally C. M. Richmond devised his crown in 1880 which for a time met all the requirements very satisfactorily. In 1885, shortly after the invention of the Richmond crown, M. C. Logan devised a crown with a large body of porcelain, having a concave counter sunk base to facilitate adaptation to the root with a dowel shaped more in line with scientific principles. More nearly approaching our requirements in conformation, form, and color, than any of its predecessors, it soon became universally adopted, and for years has had an extensive application. One objection to this crown is that the post is baked into the crown. For this purpose nothing but platinum posts could be used and they lack the necessary rigidity. To overcome this difficulty the post was made wider, baked lingually, and depressed laterally, which also is an objection on account of its weakening the root. Dr. J. G. Hollingsworth advocated the Logan crown with a band and cap by burnishing platinum foil on the basal surface of the crown, and filling the lingual V-shape space with solder. About this time the so called ''Brown Crown'' appeared, but it had a short existence. In every essential feature it was nearly like the Logan, except that the base of the crown was convex instead of concave at the expense of the root, which was a great objection. In the period of 1895-1900 the art of dentistry was making a rapid progress. It was not the protection of the root and the strength of the crown which were looked for, but rather the esthetic side of the subject. The manufactured porcelain crowns met this requirement very satisfactorily, the only disadvantage being the difficulty of fitting them to the root properly.

The Davis crown with its separable post, proved to be a more desirable crown than the Logan, Brewster, or Fellowship, designs, which had inseparable posts and consequently harder to adapt without having large root canal openings.

Let us consider the Richmond crown and bring out the advantages, disadvantages, a few important steps in its construction, and its principal indications. The Richmond crown not only protects the root, but also adds to its strength. It stands stress, forces of mastication, and meets the esthetic requirements to a certain extent. However, it has some disadvantages in certain cases. The band around the root is irritating to the gums, removing the enamel under the free margin of the gum is very painful and in some cases is very objectionable. In the anterior portion of the mouth, it does not meet the esthetic requirements as well as some other forms of crowns do because of the solder used. The dead look of the facing and showing of the gold more or less at the cervical portion often make it very undesirable. An important disadvantage is that it is not an easy crown to make. The band should always extend down to the cervical attachment of the gum, under its free margin, but not farther. On the buccal, or labial, the root should be cut down so that the cap would not show much, if any. Platinum, platinoid, and irridium posts from 14 to 18 gauge should be used, the length of which is determined according to the case. It is always perferable to have a short post than to run a risk of perforation, if there is any doubt about the case. After the cap is made, it is quite a puzzle for most of us to punch the exact position in the cap, for the post. It is suggested that a slight pressure with a round end instrument around the guessed area, will give us the right position. No matter how tight the post may fit on the cap, never take a chance of soldering it without first waxing it in position, putting a little investment inside the cap, and solder on the waxed surface with the smallest piece of solder. If the hole is punched too large, another piece of gold can be easily burnished over and soldered with the post. This does not take much time as some of you may think, but saves time. Another step which is worth mentioning, is the backing process. In soldered under crowns, the facing should be backed up the entire length, unless the bite is very heavy, while the ground in facings should be backed up to the linguo-cervical angle unless the bite is very long. A heavy single backing will do in the great majority of cases which can easily be burnished with plate burnishers. In some cases, however, the swedging process is more advisable. It always has been, and will be a difficult matter to produce the desired color with Richmond crowns. After the gold is placed on the lingual of the facing, it is quite hard to determine the extent to which the gold backing will change the color. This, of course, depending largely upon the translucency of the facing. As you have often noticed, the gold produces a dull, yellow appearance, which is very objectionable in many cases. The yellow appearance may, to an extent, be overcome by the use of platinum foil under the backing, which will neutralize, or rather produce a bluish color.

The next step, which bothers us the most, is the soldering properly without checking the facing. We have been told several times about the way of backing up the facings, and investing them. Then after the investment is more or less dry, apply the heat gradually from underneath. The point which we like to emphasize is the amount of flux necessary for this work. It seems that we have a tendency of making sure to have enough flux to flow over the margins of the facing which checks or roughens it. In cooling, it is not considered necessary to put in a cooling over or set in a dry plaster, as advocated by some, but gradual lowering of flame and letting it cool in the charcoal block is just as safe. Before we pass to the indications of Richmond, we would like to bring out in a few words, the advantages of detachable or replaceable facing variety over the soldered under. The principal advantage of it is that the cement or gutta percha used for fastening in the facing serves as a cushion, therefore in an indirect way adding to its strength. The second advantage is that it can be easily replaced if broken. This, however, should not be considered such a great advantage for the fact that this happens on account of faulty adaptation or wrong bite, which is the operator's fault. Another advantage which is brought out is the preservation of the color of porcelain by the cement which also is doubtful. In soldered under variety, this can be done by platinum foil, as already mentioned.

*Indications for a Richmond.*—The Richmond crown being the strongest of all the porcelain faced crowns, is often indicated as a bridge attachment. It is also indicated in badly broken down roots with very heavy bites. It is also indicated in older patients where the strength is the most desirable factor. The counter indications of the Richmond will be mentioned in connection with Goslee crowns.

*The Goslee Crown.*—There are two forms of Goslee crowns. One with band and cap, the other cast base. Either variety being used in badly broken roots as in the case of Richmond. The first variety is similar to the Richmond in root preparation, capping and fitting, the facing to the cap and having the linqual V-shape space cast or a plate burnished to the facing and soldered. This last method has not proved to be very satisfactory on account of the difficulty of proper burnishing of the plate to the facing. Therefore the only difference is that in the Richmond the entire occlusal, or in fact the body of the crown, is all metal, therefore stronger, while Goslee's is all porcelain, except the V-shape space on the lingual between the cap and the crown. The other variety of Goslee is that of a cast base which has been more of a puzzle to most of us than all the other crowns put together. The cast base Goslee differs from that of ordinary cast base in one point. The Goslee crown has a step on the lingual extending half way mesio-distally for the purpose of retention, additional strength to the base without much loss of the porcelain body and a location for the sprue. The cast base Goslee having a large body of porcelain is more translucent, vital, natural, esthetic, and in some cases more hygienic or less irritating than the Richmond. We are living in an age where these features are considered more than the mere strength. No one will be excused for inserting a Richmond crown on the six anteriors, or even on the bicuspids, except in the three instances which we already have indicated in the discussion of the Richmond crown: such as bridge attachments, old age, and very heavy bite. This fact can not be emphasized too much, because the tendency seems to be to insert Richmonds any place where there is a broken root.

It may not be out of place to say a word about temporary crowns, which may have an occasional practical use for us which will be realized when the case presents. This is made by fitting the facing over the root the best possible in a short time; then set a post in the root canal, and simply cement the facing to the post with temporary cement, or gutta percha. Or, if strength is desired, the facing can be backed up and soldered to the post which can be done in a few minutes.

The extirpation of vital pulps in the six anterior teeth which have single root canals, is easily done by the high pressure or low pressure injection method as the case indicates. As a rule, all the posterior teeth should be devitalized by arsenic. The advantage of arsenic is due to the fact that it fully destroys the pulp which is very desirable in unaccessible cases and in cases where we have very irregular roots. It is not necessary for us to repeat all the minor but important details which have been emphasized so often. A few points should always be kept in mind. Be sure and remove all the fibrils in high or low pressure method, at the first sitting, because if you do not, you will have quite a time in removing them the next sitting. Be careful not to perforate the apex because the tissues will be more or less desensitized and the patient will not be a good guide. Do not fill the root canals until all the soreness disappears after the removal of the pulp. In taking all these precautions, we do not waste but save time and trouble. We also have to be very careful in not injuring the gums or the peridental membrane, which may cause a long siege of soreness or an abscess.

In conclusion we say that with all the knowledge we have, if used conscientiously, that is, if the highest standard and the better service of men is considered over time and money, we are fully confident that the art of dentistry will be brought up to that level which is the most respected ideal of the present day professional man.

